# Supplementary material for: Influence of Mechanical Fatigue at Different States of Charge on Pouch-Type Li-Ion Batteries
Source: Materials (Basel). 2022 Aug 12;15(16):5557. doi: 10.3390/ma15165557 (PMC9413291; doi:10.3390/ma15165557)
Supplement: Supplementary file 1 [file materials-15-05557-s001.zip › materials-1795479-supplementary.pdf]

materials

MDPI

Supplementary Materials

Influence of Mechanical Fatigue at Different States of Charge on Pouch-Type Li-Ion Batteries

Jin-Yeong Kim [1,†], Jae-Yeon Kim [2,†], Yu-Jin Kim [1], Jaeheon Lee [2], Kwon-Koo Cho [3], Jae-Hun Kim [4,*] and Jai-Won Byeon [1,*]

[1] Department of Materials Science and Engineering, Seoul National University of Science and Technology, Seoul 01811, Korea
[2] Department of Mining and Geological Engineering, University of Arizona, Tucson, AZ 85721, USA
[3] Department of Materials Engineering and Convergence Technology, Research Institute for Green Energy Convergence Technology, Gyeongsang National University, 501, Jinju-daero, Jinju-Si 52828, Korea
[4] School of Materials Science and Engineering, Kookmin University, Seoul 02707, Korea
[*] Correspondence: jaehunkim@kookmin.ac.kr (J.-H.K.); byeonjw@seoultech.ac.kr (J.-W.B.); Tel.: +82-2-910-5740 (J.-H.K.); +82-2-970-6634 (J.-W.B.)
† These authors contributed equally to this work.

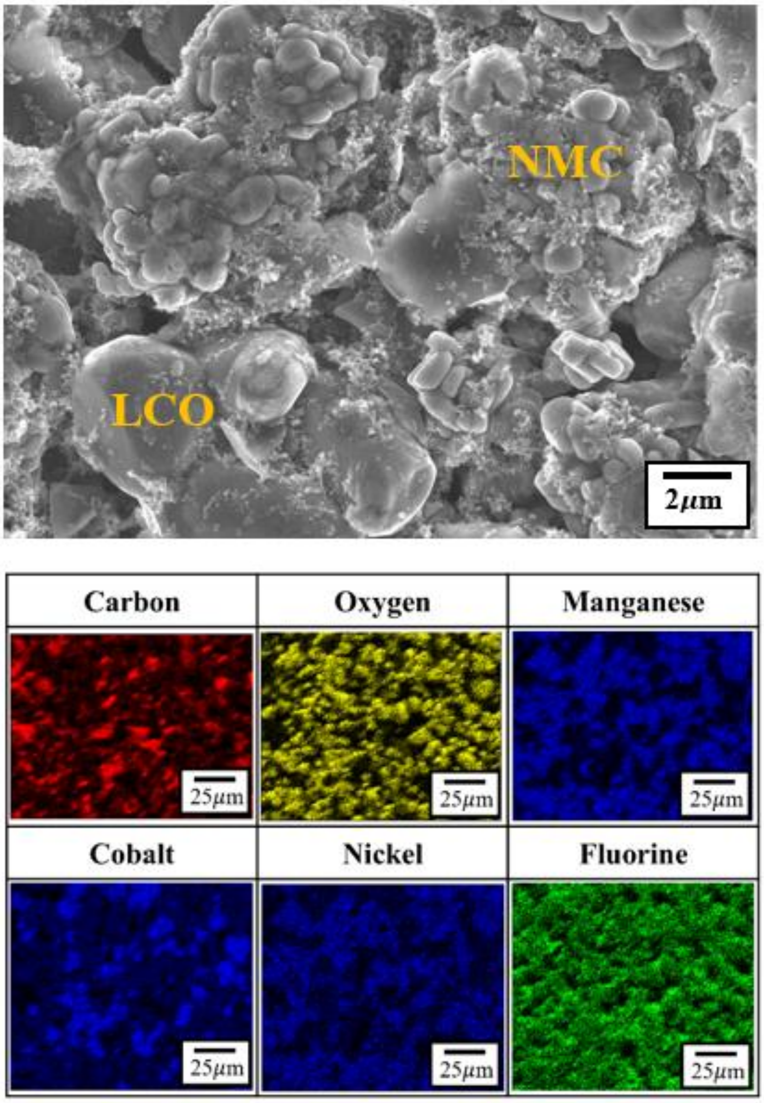

Element	C	O	Ni	Co	Mn	F
Weight (%)	21.9	20.9	10.3	25.8	10.3	10.8

Figure S1. FE-SEM and EDS elemental mapping results for the LCO/NCM cathode. The below table shows the result of EDS mapping analysis.

Table S1. Inductively coupled plasma (ICP) analysis result for the LCO/NCM cathode.

Element	Li	Ni	Co	Mn
Weight (%)	8.00	13.0	67.3	11.7

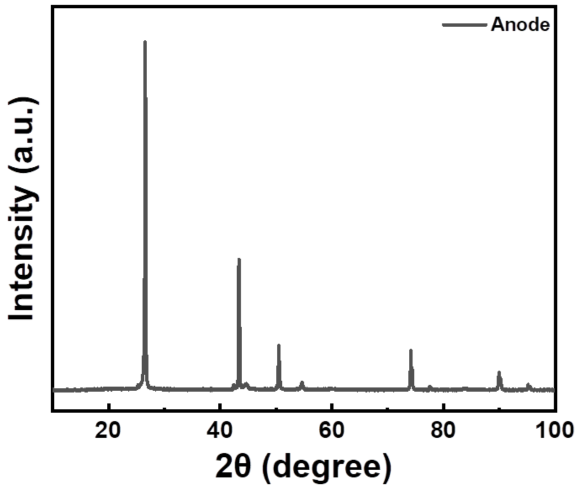

Figure S2. X-ray diffraction pattern (XRD) of the graphite anode.

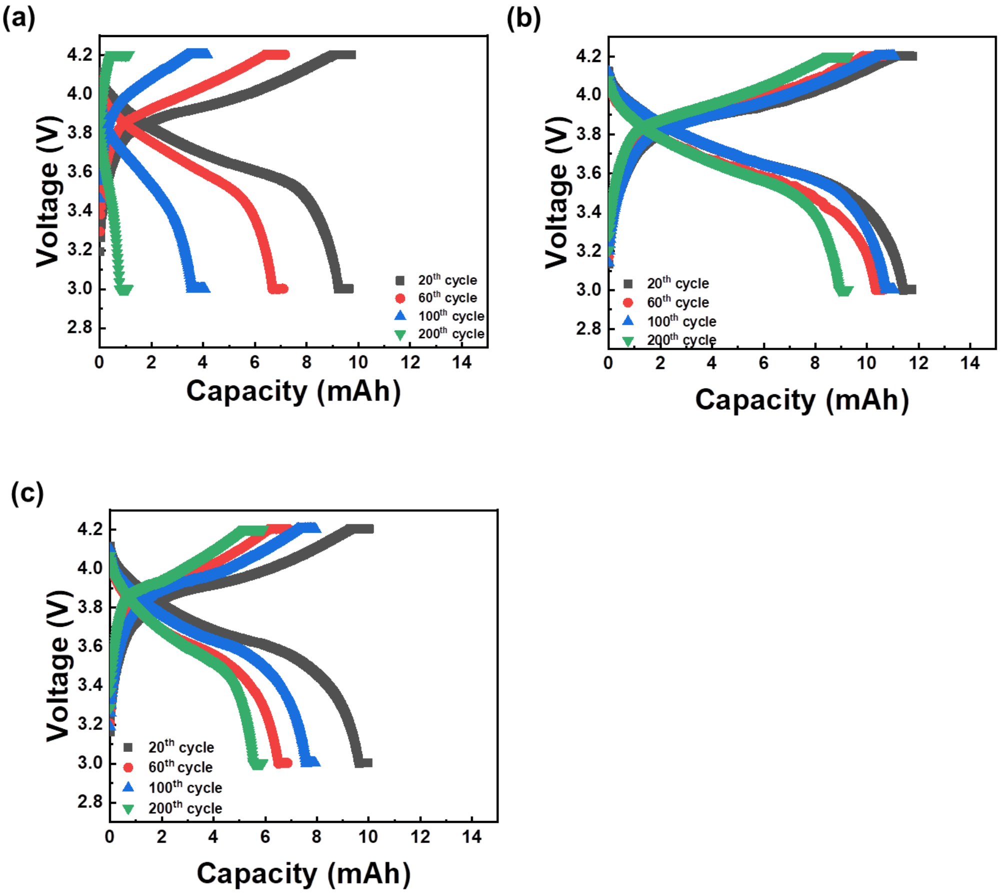

Figure S3. Charge and discharge voltage profiles of the cells after the mechanical fatigue test: (**a**) SoC 60% cell, (**b**) SoC 70% cell, and (**c**) SoC 100% cell.

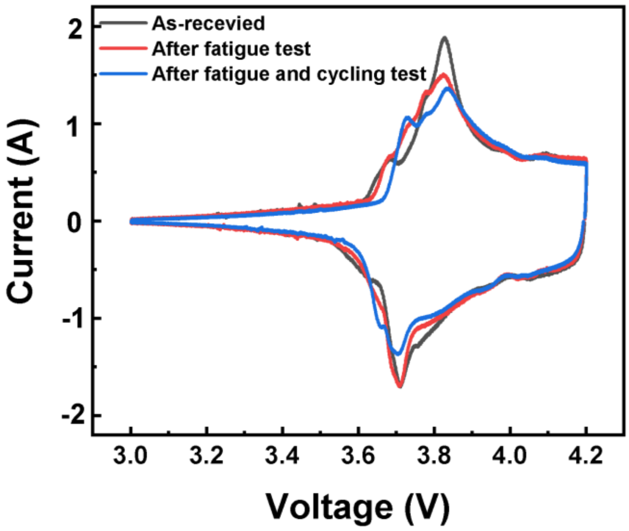

Figure S4. Cyclic voltammograms of the cell (initial SoC of 70%) under different test conditions (as-received, after the fatigue test, and after the fatigue and cycling test).

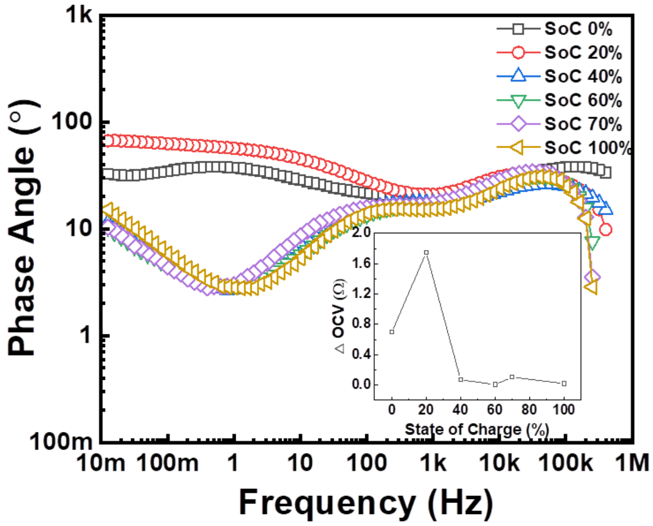

Figure S5. Bode plots of the cells with different initial SoCs immediately after the mechanical fatigue test.
